# Fabrication of InGaN thin-film transistors using pulsed sputtering deposition

**DOI:** 10.1038/srep29500

**Published:** 2016-07-07

**Authors:** Takeki Itoh, Atsushi Kobayashi, Kohei Ueno, Jitsuo Ohta, Hiroshi Fujioka

**Affiliations:** 1 Institute of Industrial Science, The University of Tokyo, Tokyo 153-8505, Japan; 2 PRESTO, Japan Science and Technology Agency, Saitama 332-0012, Japan; 3 ACCEL, Japan Science and Technology Agency, Tokyo 102-0075, Japan

## Abstract

We report the first demonstration of operational InGaN-based thin-film transistors (TFTs) on glass substrates. The key to our success was coating the glass substrate with a thin amorphous layer of HfO_2_, which enabled a highly *c*-axis-oriented growth of InGaN films using pulsed sputtering deposition. The electrical characteristics of the thin films were controlled easily by varying their In content. The optimized InGaN-TFTs exhibited a high on/off ratio of ~10^8^, a field-effect mobility of ~22 cm^2^ V^−1^ s^−1^, and a maximum current density of ~30 mA/mm. These results lay the foundation for developing high-performance electronic devices on glass substrates using group III nitride semiconductors.

Thin-film transistors (TFTs) are key devices in large-area electronics and are fabricated on various types of amorphous substrates, such as glass or plastic^1^,^2^,^3^. Although hydrogenated amorphous silicon (a-Si:H)^4^ and amorphous oxide semiconductors such as InGaZnO (IGZO)^5^,^6^,^7^ are widely used as channel materials in TFTs, the electron mobility of these materials falls short of realizing high-performance electronic systems fabricated on amorphous materials. Conversely, group III nitride semiconductors, which have already been used as commercial transistors in ultrafast communication systems^8^,^9^, are promising materials for high-performance TFTs if they can be successfully accommodated on large-area amorphous substrates. In industrial applications, however, film growth of nitride semiconductors is usually achieved using metal-organic vapor-phase epitaxy^10^,^11^ at approximately 1000 °C^12^,^13^,^14^ in order to enhance the chemical reactivity of the raw materials. This temperature is obviously incompatible with large-area flat glass substrates. In order to overcome this problem and fabricate TFTs on conventional glass substrates, a novel growth technique that allows us to obtain high-quality nitride semiconductors on glass at low temperatures must be developed. Recently, the use of a new growth technique called pulsed sputtering deposition (PSD) was proposed for the preparation of group III nitrides^15^. During growth with PSD, high-energy film precursors formed in a plasma help produce high-quality group III nitride materials even at low temperatures (e.g., lower than 500 °C); this enables us to obtain InGaN films without phase separation and/or to employ thermally unstable substrates such as glass. In fact, we fabricated InGaN-based full-color light emitting diodes on graphene-covered amorphous SiO_2_ surfaces^16^. In this process, graphene was utilized as a buffer layer to promote *c*-axis orientation of the InGaN layers. Unfortunately, this orienting buffer layer is so conductive that it also acts as a leakage pathway when used as a substrate for TFTs. Therefore, fabrication of nitride-based TFTs on glass substrates requires an alternative orienting buffer layer of a highly insulating nature. In this study, we investigated the use of HfO_2_ as an orienting layer for InGaN film growth on glass substrates. We also explored the feasibility of producing InGaN-based TFTs on large-area amorphous substrates using a low-temperature PSD growth technique.

Figure 1 shows XRD patterns of the InGaN films grown on glass substrates in this study. An In_*x*_Ga_1−*x*_N film with *x* = 0.5, directly grown on a glass substrate, did not show any diffraction peaks because of poor crystallinity. Conversely, the InGaN films grown on HfO_2_-coated glass substrates exhibited peaks attributed to 0002 diffraction. The appearance of a sole 0002 diffraction peak in the XRD patterns indicates that highly *c*-axis-oriented InGaN films grow on HfO_2_-coated glass substrates. The full width at half maximum values of rocking curves for 0002 diffraction were larger than 1° for all samples. It was impossible to detect asymmetric diffraction of the InGaN films due to poor in-plane alignment. The In contents of the InGaN films prepared on the HfO_2_ buffer indicated in Fig. 1 are derived from the peak positions of the diffractions assuming that Vegard’s law is valid. Reflection high-energy electron diffraction observations (not shown) revealed that the InGaN films grown on HfO_2_-coated glass substrates are polycrystalline. An atomic force microscopic image of an In_0.30_Ga_0.70_N film grown on a HfO_2_-coated glass substrate is shown in Fig. 2. Crystal grains with a size of ~10 nm are homogeneously grown on the substrate. The root-mean-square roughness of the InGaN surface was 1.8 nm, which is smooth enough to be used as a channel in a field-effect transistor. Coating glass substrates with HfO_2_ is also important for obtaining InGaN films with smooth surfaces and for suppressing electron scattering caused by rough interfaces between channel and gate dielectrics.

Herein, 50 μm × 50 μm square mesas of 80-nm-thick *c*-axis-oriented InGaN were formed using an inductively coupled plasma (ICP) dry etching technique. Ti/Al electrodes were deposited on the corners of the InGaN mesas to perform van der Pauw Hall-effect measurements. The In content of the InGaN was evaluated with energy-dispersive X-ray spectroscopy (EDS). Figure 3 summarizes the relationship between the In composition, electron concentration, and electron mobility of the InGaN films. Note that since the Hall voltages for InGaN with In content *x* less than 0.6 were too low to be detected using our Hall-effect measurement setup, it was impossible to calculate the electron mobility and concentration of those InGaN. As the In composition decreases, the electron concentration of the InGaN monotonically decreases. An InGaN film with an In content of *x* = 0.60 has an electron concentration of ~1 × 10^19^ cm^−3^, whereas the electron concentration of a pure InN film is over 1 × 10^20^ cm^−3^. This result indicates that the electron concentration in InGaN films can be controlled by changing the In content. It is well known that as the In content in InGaN decreases, the Fermi-level stabilization energy drops from the conduction band into the forbidden gap^17^,^18^, which possibly explains the decrease in the electron concentration. Figure 3 also shows that the electron mobility of InGaN films decreases as the In content decreases. One of the reasons for this decrease is alloy scattering. Other possible explanations are the decrease in electron effective mass in InGaN with decreased In content and the formation of an energy barrier at the InGaN grain boundaries where the conduction band is possibly bent upward^19^.

TFTs with InGaN channels were fabricated by photolithography and Cl_2_ ICP dry etching technologies. First, InGaN films were etched into 200 μm × 50 μm mesa structures. Source and drain electrodes (Ti/Al: 10 nm/30 nm) were subsequently deposited by sputtering, and a-HfO_2_ gate dielectrics were deposited on the InGaN films by atomic layer deposition (ALD) at 200 °C. Finally, gate electrodes (Au: 50 nm) were deposited by vacuum deposition. An optical microscope image and a schematic drawing of the transistor are shown in Fig. 4.

Figure 5 shows the field-effect mobility of the InGaN TFTs. It is clear that the mobility strongly depends on the In content of the InGaN channel layer. As the In composition decreases, the mobility decreases from 4 to 0.16 cm^2^ V^−1^ s^−1^ (indicated as Region I), but rapidly increases to 22 cm^2^ V^−1^ s^−1^ at an In composition of 0.45 (Region II), before again decreasing to <1 cm^2^ V^−1^ s^−1^ (Region III). The field-effect mobility of transistors is determined by several factors, such as the carrier mobility in the film, the electron concentration in the channel, and the quality of the interfaces between the InGaN and the dielectrics. 20-nm-thick HfO_2_ (*ε*_r_ = 15) with a bias voltage of 5 V can potentially accumulate (or deplete) sheet electrons of ~2 × 10^13^ cm^−2^, which correspond to the electron density of 4 × 10^19^ cm^−3^ for 50-nm-thick InGaN. In Region I, the field-effect mobility is mainly affected by carrier mobility since the electron concentration is almost constant (Fig. 3). We speculate that the electron concentration rapidly decreased in Region II. (In fact, it cannot be measured due to the detection limit of our Hall-effect measurement). Figure S1 (Supplementary Information) shows the dependence of ON current in InGaN TFTs on In content. In Region II, the ON current falls ~10^−2^. If we assume that the carrier concentration closely tied with the ON current, we could conclude that carrier concentration of In_0.45_Ga_0.55_N is on the order of ~10^17^ cm^−3^, which can be adequately controlled with gating using the 20-nm-thick HfO_2_. We believe that the rapid decrease in electron concentration in this region strongly affected the field-effect mobility compared to decrease in the electron (Hall) mobility. In Region III, the carrier concentration decreases further, and the contact resistance between InGaN and Ti/Al electrodes probably increases, which results in lowering the ON current (Fig. S1). The electron mobility also decreased. These factors lead to the lowered field-effect mobility in Region III. Figure 6 shows (a) the output and (b) the transfer characteristics of the In_0.45_Ga_0.55_N TFT, which exhibited the best performance in this study. The on/off ratio of the TFT was as high as ~10^8^, and the field-effect mobility in the saturation region, *μ*_sat_, was determined to be 22 cm^2^ V^−1^ s^−1^. Moreover, the current density in the on-state was as high as 30 mA/mm, which is obviously higher than in conventional TFTs employing a-Si:H or IGZO. Note that the surface of In_0.45_Ga_0.55_N film (Fig. S2) exhibiting the best TFT performance is as flat as that of In_0.30_Ga_0.70_N film.

In summary, we have succeeded in fabricating operational InGaN-based TFTs on glass substrates for the first time. We found that highly *c*-axis-oriented InGaN films can be grown by PSD on glass coated with amorphous HfO_2_ layers. Hall effect measurements have revealed that the electron concentration of the *c*-axis-oriented InGaN on glass can be controlled by varying their In content. An InGaN-based TFT with an In concentration of 0.45 exhibited the highest on/off ratio of ~10^8^, a field-effect mobility of ~22 cm^2^ V^−1^ s^−1^, and a maximum current density of ~30 mA/mm. These excellent TFT characteristics prove that nitride semiconductors are promising materials for realizing high-performance electronic devices on glass substrates.

## Methods

InGaN films were grown by PSD^15^,^16^,^20^,^21^ and their In content was modulated by changing the supply ratio of In and Ga. Before the growth, fused silica glass substrates were degreased by ultrasonic cleaning in ethanol for 5 min. InGaN films were grown on the glass substrates, with and without an amorphous HfO_2_ layer deposited by ALD at 200 °C. Tetrakis(dimethylamido)hafnium (Hf(NMe_2_)_4_) and O_3_ were used as precursors for the amorphous HfO_2_. The growth of InGaN was carried out at 300 °C. After the growth, metal droplets remaining on the surface of the InGaN films were immediately removed using HCl (~35%) solution. The InGaN samples were cut into specimens with a size of 10 mm × 10 mm and subjected to the XRD measurements.

## Additional Information

**How to cite this article**: Itoh, T. *et al*. Fabrication of InGaN thin-film transistors using pulsed sputtering deposition. *Sci. Rep.* **6**, 29500; doi: 10.1038/srep29500 (2016).

## Supplementary Material

Supplementary Information

## Figures and Tables

**Figure 1:**
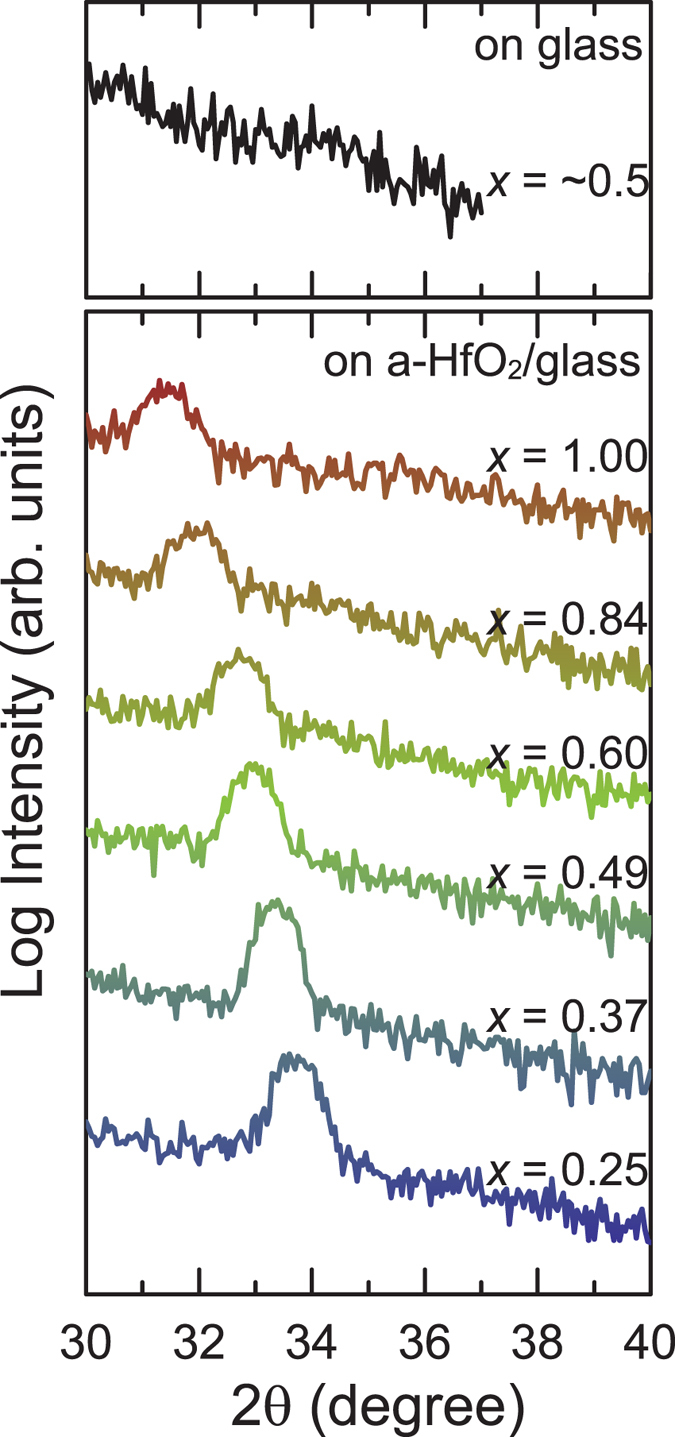
XRD 2θ scans of In_x_Ga_1−*x*_N films with various In contents (*x*) grown on glass (upper) and a-HfO_2_-coated glass (lower). The In content indicated in the lower graph was calculated using Vegard’s law.

**Figure 2:**
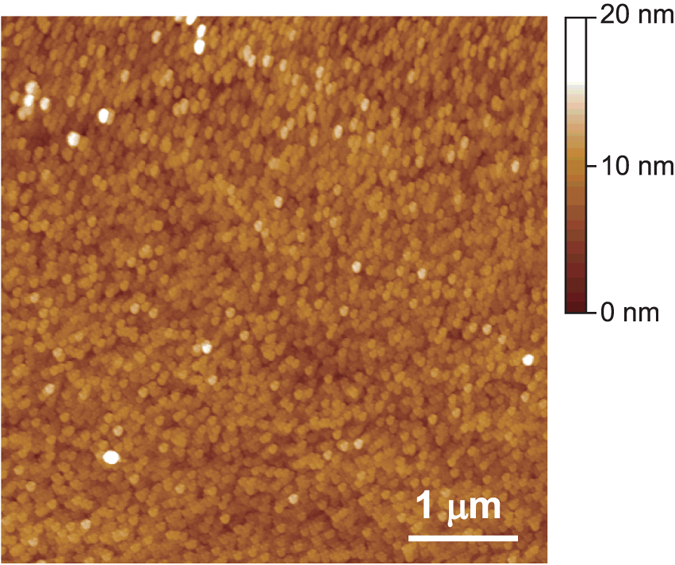
Atomic force microscope image of an In_0.30_Ga_0.70_N film grown on an a-HfO_2_-coated glass substrate. The root-mean-square surface roughness was 1.8 nm, and the average grain size was ~10 nm.

**Figure 3:**
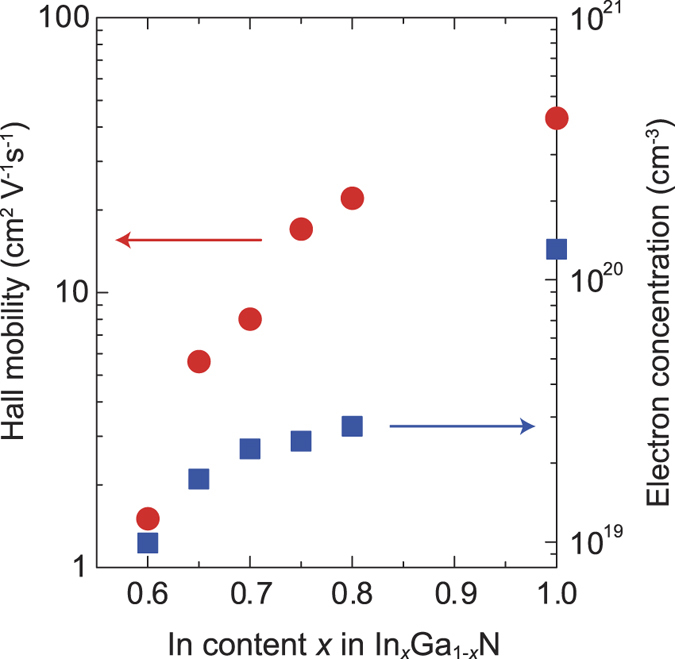
Electron mobility and concentration of *c*-axis-oriented In_x_Ga_1−*x*_N films on glass determined by Hall effect measurements.

**Figure 4:**
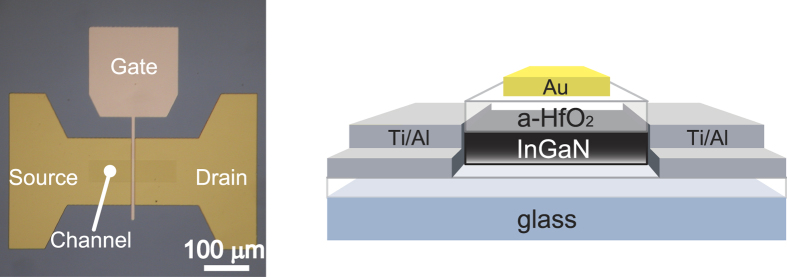
Optical microscopic image and schematic drawing of an InGaN-based thin-film transistor.

**Figure 5:**
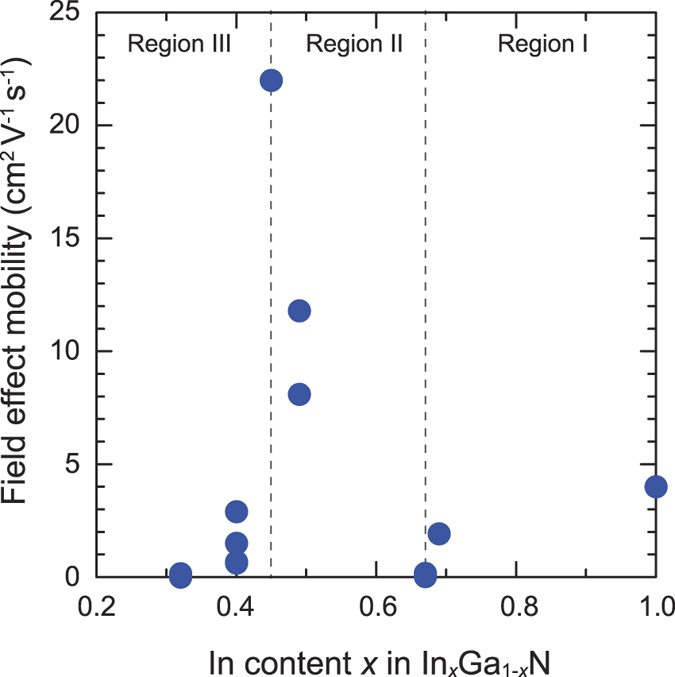
Field-effect mobility of In_x_Ga_1−*x*_N thin-film transistors. The In compositions were determined from the EDS analysis on the InGaN mesas fabricated close to the FETs. The graph is divided into three regions. The mobility changes in each region are explained in the text.

**Figure 6:**
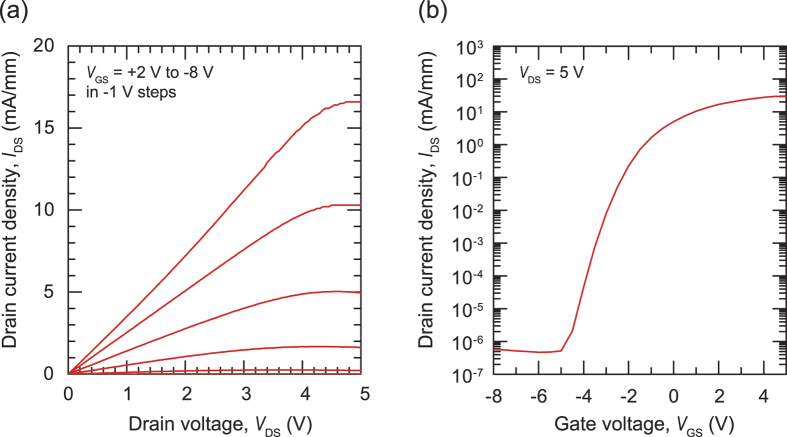
(**a**) Output and (**b**) transfer characteristics of an In_0.45_Ga_0.55_N thin-film transistor.
