# Supplementary material for: Fabrication of InGaN thin-film transistors using pulsed sputtering deposition
Source: Sci Rep. 2016 Jul 7;6:29500. doi: 10.1038/srep29500 (PMC4935987; doi:10.1038/srep29500)
Supplement: Supplementary Information [file srep29500-s1.doc]

**Fabrication of InGaN thin-film transistors
using pulsed sputtering deposition**

**Supplementary Information**

Takeki Itoh,^1^ Atsushi Kobayashi,^1^ Kohei Ueno,^1^ Jitsuo Ohta,^1,2^ and Hiroshi Fujioka^1,3,*^

*^1^Institute of Industrial Science, The University of Tokyo, Tokyo 153-8505, Japan*

*^2^PRESTO, Japan Science and Technology Agency, Saitama 332-0012, Japan*

*^3^ACCEL, Japan Science and Technology Agency, Tokyo 102-0075, Japan*

**Dependence of ON current of InGaN TFTs on In content**

Fig. S1 The dependence of ON current in InGaN TFTs on In content. The channel length and width are 10 and 50 μm, respectively.

**Surface morphology of In_0.45_Ga_0.55_N grown on an amorphous-HfO_2_-coated glass**


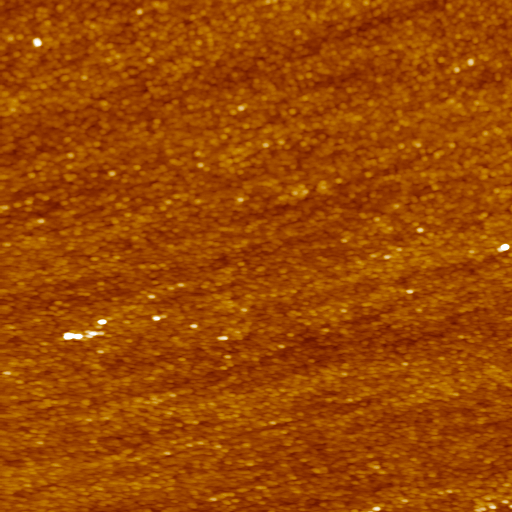


Fig. S2 Atomic force microscope image (5 μm × 5 μm) of an In_0.45_Ga_0.55_N film grown on an a-HfO_2_-coated glass substrate. The root-mean-square surface roughness was 1.2 nm.
